# Machine Learning Predictive Models for Evaluating Risk Factors Affecting Sperm Count: Predictions Based on Health Screening Indicators

**DOI:** 10.3390/jcm12031220

**Published:** 2023-02-03

**Authors:** Hung-Hsiang Huang, Shang-Ju Hsieh, Ming-Shu Chen, Mao-Jhen Jhou, Tzu-Chi Liu, Hsiang-Li Shen, Chih-Te Yang, Chung-Chih Hung, Ya-Yen Yu, Chi-Jie Lu

**Affiliations:** 1 Division of Urology, Department of Surgery, Far Eastern Memorial Hospital, New Taipei City 220, Taiwan; 2 Department of Healthcare Administration, College of Healthcare & Management, Asia Eastern University of Science and Technology, New Taipei City 220, Taiwan; 3 Graduate Institute of Business Administration, Fu Jen Catholic University, New Taipei City 242, Taiwan; 4 Department of Business Administration, Tamkang University, New Taipei City 251, Taiwan; 5 Department of Laboratory Medicine, Taipei Hospital, Ministry of Health and Welfare, New Taipei City 242, Taiwan; 6 Department of Medical Laboratory, Chang-Hua Hospital, Ministry of Health and Welfare, Chang Hua County 513, Taiwan; 7 Artificial Intelligence Development Center, Fu Jen Catholic University, New Taipei City 242, Taiwan; 8 Department of Information Management, Fu Jen Catholic University, New Taipei City 242, Taiwan

**Keywords:** health screening indicator, machine learning, male reproductive health, sperm quality, sleep time

## Abstract

In many countries, especially developed nations, the fertility rate and birth rate have continually declined. Taiwan’s fertility rate has paralleled this trend and reached its nadir in 2022. Therefore, the government uses many strategies to encourage more married couples to have children. However, couples marrying at an older age may have declining physical status, as well as hypertension and other metabolic syndrome symptoms, in addition to possibly being overweight, which have been the focus of the studies for their influences on male and female gamete quality. Many previous studies based on infertile people are not truly representative of the general population. This study proposed a framework using five machine learning (ML) predictive algorithms—random forest, stochastic gradient boosting, least absolute shrinkage and selection operator regression, ridge regression, and extreme gradient boosting—to identify the major risk factors affecting male sperm count based on a major health screening database in Taiwan. Unlike traditional multiple linear regression, ML algorithms do not need statistical assumptions and can capture non-linear relationships or complex interactions between dependent and independent variables to generate promising performance. We analyzed annual health screening data of 1375 males from 2010 to 2017, including data on health screening indicators, sourced from the MJ Group, a major health screening center in Taiwan. The symmetric mean absolute percentage error, relative absolute error, root relative squared error, and root mean squared error were used as performance evaluation metrics. Our results show that sleep time (ST), alpha-fetoprotein (AFP), body fat (BF), systolic blood pressure (SBP), and blood urea nitrogen (BUN) are the top five risk factors associated with sperm count. ST is a known risk factor influencing reproductive hormone balance, which can affect spermatogenesis and final sperm count. BF and SBP are risk factors associated with metabolic syndrome, another known risk factor of altered male reproductive hormone systems. However, AFP has not been the focus of previous studies on male fertility or semen quality. BUN, the index for kidney function, is also identified as a risk factor by our established ML model. Our results support previous findings that metabolic syndrome has negative impacts on sperm count and semen quality. Sleep duration also has an impact on sperm generation in the testes. AFP and BUN are two novel risk factors linked to sperm counts. These findings could help healthcare personnel and law makers create strategies for creating environments to increase the country’s fertility rate. This study should also be of value to follow-up research.

## 1. Introduction

Population aging is one of the by-products of a country’s economic development. It increases the burden on the younger generation and diminishes the time available to raise the next generation. Fertility and birth rates have been continually declining in many countries. Taiwan’s fertility rate reached its lowest point in 2022 at 1.08 children born per woman, which is lower than the 2.1 needed to maintain the population [1]. Therefore, it is crucial to ensure that married couples wanting to raise the next generation are able to conceive successfully. However, around 15–20% of couples are unable to conceive within one year of unprotected intercourse. Male factors contribute to 50% of all infertile cases. Although advances in assisted reproductive techniques (ART) help many couples to conceive successfully, the success rate of ARTs still depends on semen quality [2].

The decline in fertility has coincided with the falling trend in semen quality in recent years. Sperm count and sperm concentration, two determinants of semen quality, were found to be declining in a meta-analysis of 61 studies published between 1938 and 1990 comparing men with no history of infertility [3]. Since that finding, multiple studies have confirmed this worrying trend of decreasing sperm count and sperm density. In a more recent study, the proportion of men with normal total motile sperm count (>15 million) was found to have declined by about 10% over the past 16 years [4]. Although this trend was found within the subfertile male population, it implies that more couples need ARTs to help them to conceive.

Many risk factors, ranging from the patient’s genetic background [5], maternal exposure [6], environmental pollutants [7], metabolic syndrome (MetS) [8], and obesity [9] to the patient’s lifestyle [10], have been recognized to affect sperm count. Sperm count is further associated with sperm quality and could determine male fertility [11]. However, the extent of the influence of these factors on semen quality remains to be clearly determined due to the inability to design an experiment to account for all possible confounding factors. In addition, many previous study populations were recruited from infertility centers and their conclusions were not representative of the general population. Therefore, to gain more insights into the interplay between these factors and male fertility in the general population, we are the first study to analyze the annual health screening data, the MJ health-check-up-based population database (MJPD), from a major health screening center in Taiwan. The MJPD is widely used in the healthcare/medical informatics studies [12]. Patients with metabolic syndrome, hyperlipidemia, or different lifestyles were considered and used in this study to analyze the impacts of these risk factors on semen count.

Most of the existing studies usually utilized traditional multiple linear regression (MLR) to analyze the relationship between risk factors and sperm count [13,14,15]. MLR assumes that the dependent variable should be linearly correlated with independent variables and that collinearity should not occur between independent variables [16,17,18]. However, the use of MLR has limitations when the data may have non-linear relationships or complex interactions between variables [16]. Machine learning (ML) methods are data-driven algorithms and do not require statistical assumptions. They can capture non-linear relationships between variables or those with complex interactions [19,20,21,22]. As ML methods can handle collinearity more effectively than MLR and generate promising performances, they have been widely used for prediction issues in the field of healthcare/medical informatics, while MLR is used as a baseline for comparison [23,24,25,26]. However, only a few studies have utilized ML for sperm-count-related research [27,28,29].The five effective ML methods with different modeling mechanisms, namely, random forest (RF), stochastic gradient boosting (SGB), least absolute shrinkage and selection operator regression (Lasso), ridge regression (Ridge), and extreme gradient boosting (XGBoost), are used in this study since they have been successfully utilized in many healthcare or medical informatics studies to provide promising results [24,25,30,31,32,33,34,35,36,37,38,39]. Thus, this study aims to construct a framework based on RF, SGB, Lasso, Ridge, and XGBoost prediction models to identify the major risk factors affecting male sperm count in order to provide more sperm-count-related research that utilizes ML in the field of reproductive biology.

## 2. Materials and Methods

### 2.1. Data Material

The process for identifying subjects in this study consisted of scrutinizing health screening indicators and questionnaire records of 71,108 members of the MJPD for the period 2005–2017. The study selected 30 health screening indicators and questionnaire variables relevant to the investigation. As there might have been multiple annual screening data for each member in the database, only the most recent annual record of the subject was analyzed. Subjects who lacked data on the main study variables were excluded, leaving 30,255 individuals who met the study eligibility criteria. We excluded 6 subjects who were older than 50 years and not evenly distributed in the study groups and 28,874 non-male subjects for whom sperm counts or motility tests were not performed in their annual health examination. We finally identified 1375 eligible male subjects, of whom 686 (49.89%) were married and 619 (45.02%) were unmarried, with an average age of 33.22 ± 4.36 years.

In Taiwan, many studies using the MJPD are listed on the website (http://www.mjhrf.org/main/page/resource/en/#resource07; accessed on 1 October 2022). The MJPD includes data collected from four MJ clinics that provide health screening to the center’s members. All the datasets used were authorized by MJ Health Research Foundation (Approval No.: MJHRF-2016005A). The data application procedures are described at http://www.mjhrf.org/main/page/release1/en/#release01(accessed on 1 October 2022). The MJPD is accessible to academic researchers upon request. The protocol of this study was evaluated for ethical issues regarding the use of data in the database and was deemed acceptable by the Research Ethics Review Committee of Far Eastern Memorial Hospital (FEMH-IRB-107127-E, Protocol Version 1, 15 February 2022) and the MJ Health Research Foundation; it was approved by ClinicalTrials.gov (ID: NCT05225454). The study was conducted according to the guidelines of the Declaration of Helsinki, and all data were anonymized before analysis in accordance with the ethics requirements of the institutional review board.

**Figure 1:**
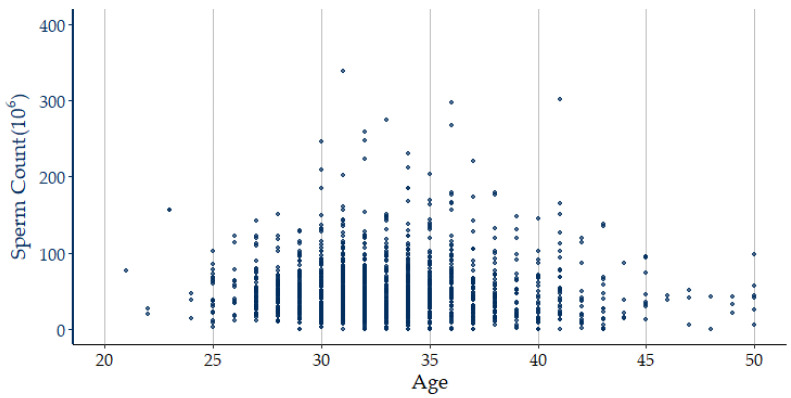
Sperm count distribution by age.

**Figure 2:**
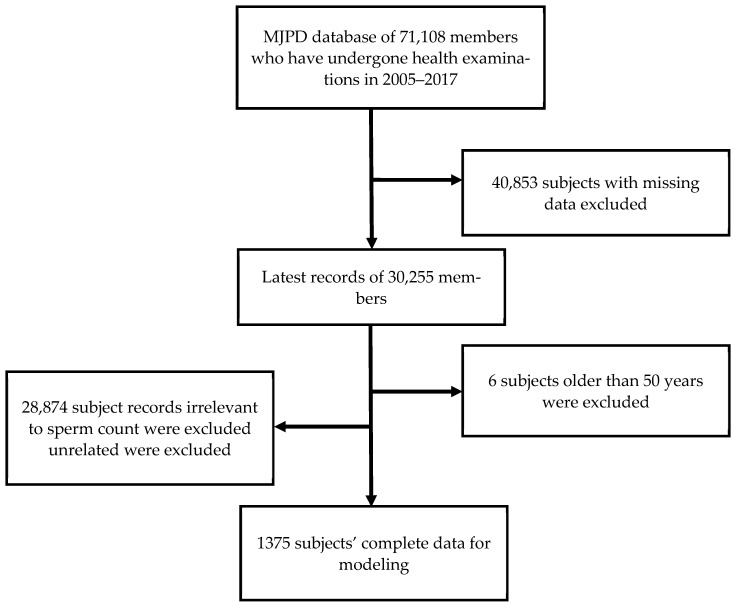
Subject identification process.

**Figure 3:**
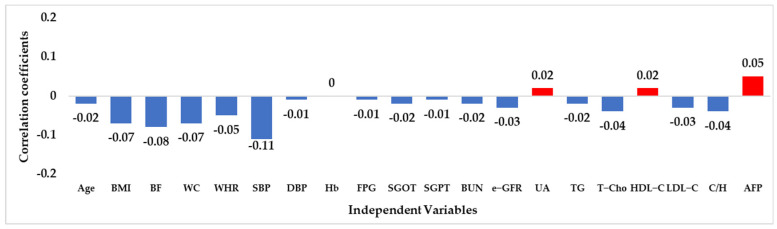
Correlation coefficients between numerical independent variables and sperm count. Note: BMI: body mass index; BF: body fat; WC: waist circumference; WHR: waist–hip ratio; SBP: systolic blood pressure; DBP: diastolic blood pressure; Hb: hemoglobin; FPG: fasting plasma glucose; SGOT: serum glutamic oxaloacetic transaminase; SGPT; serum glutamic pyruvic transaminase; BUN: blood urea nitrogen; e-GFR: estimated glomerular filtration rate; UA: uric acid; TG: triglyceride; T-Cho: total cholesterol; HDL-C: high-density lipoprotein cholesterol; LDL-C: low-density lipoprotein cholesterol; C/H: T-Cho/HDL-C; AFP: alpha–fetoprotein.

Figure 1 illustrates the sperm count distribution in different age groups in the sample, while Figure 2 shows the subject identification process for selecting the sample in this study. Table 1 provides the sample attributes of the subjects, including descriptive statistics of the independent and dependent variables. Figure 3 presents the correlation coefficients between 20 numerical independent variables and sperm count using Pearson correlation analysis. It can be seen from Figure 3 that a total of 3 risk factors have a positive linear correlation with the dependent variable, namely, UA, HDL-C, and AFP. A total of 16 risk factors have a negative linear correlation with the dependent variable, namely Age, BMI, BF, WC, WHR, SBP, DBP, Hb, FPG, SGOT, SGPT, BUN, e-GFR, TG, T-Cho, LDL-C, and C/H. Hb has no linear correlation with the dependent variable. Although all of the numerical independent variables do not have a strong linear correlation with the dependent variable, there may be non-linear relationships or complex interactions between variables. Therefore, the five ML predictive algorithms were used in this study as they can analyze data with non-linear relationships or complex interactions between variables [19,20,21,22].

### 2.2. Proposed Framework

**Figure 4:**
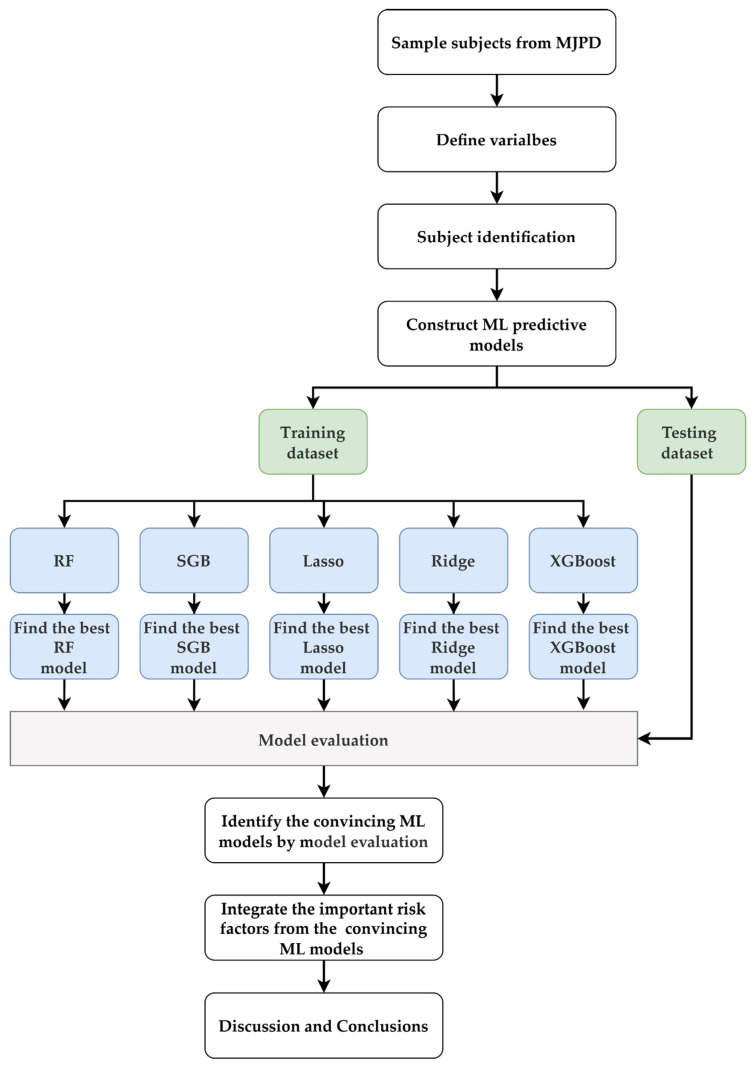
Proposed framework based on machine learning prediction models. Note: MJPD: MJ health-check-up-based population database; ML: machine learning; RF: random forest; SGB: stochastic gradient boosting; Lasso: least absolute shrinkage and selection operator regression; Ridge: ridge regression; XGBoost: extreme gradient boosting.

In this study, a framework was constructed using the five ML prediction models for the identification of important risk factors (independent variables) affecting sperm count, integration, and deliberation. The proposed ML prediction model-based risk factor evaluation framework is shown in Figure 4. 

In the proposed framework, the first step involved selecting subjects from the MJPD for the analysis. In the second step, candidate risk variables were chosen and target variables were defined. Twenty-nine risk factors were used as predictor (independent) variables and sperm count was the target (dependent) variable. In the third step, the sperm count of each subject was identified. After the data were organized, the fourth step involved construction of the prediction model for sperm count using the five ML techniques: RF; SGB; Lasso; Ridge; and XGBoost.

RF is a technique that integrates decision tree methods [40]. It randomly generates multiple different and unpruned decision trees, each of which determines the growth of the tree based on the Gini index, and integrates all the trees generated into a forest. It then averages or votes for the trees in the forest to produce a stable ensemble model, thereby reducing correlation between trees and generalization error. Eventually, a stable ensemble model is generated. SGB implements a combination of bagging and boosting [41,42] to generate numerous additive regression trees by multiple iterations. Each tree is trained according to the residuals of the previous iteration [42]. The final number of additive regression trees is determined by satisfying the maximum number of iterations or the convergence condition. Finally, the cumulative result of multiple trees is obtained by weighted summation to determine the final stable model.

Lasso is an extension of the conventional regression method and is based on the principle of using the least absolute shrinkage and selection operator (L1 regularization) to reduce the overfitting problem by forcing the coefficients that contribute less variance to the model to exactly zero, thereby obtaining a lower variance [43,44]. Ridge has the same basic concept as Lasso, with the main difference being that Ridge uses L2 regularization to reduce the coefficients in the model. Ridge adds an appropriate L2 penalty to the model to reduce all coefficients to non-zero values or values close to zero, and then minimizes the sum of squared errors to further control the trade-off between bias and variance to reduce overfitting [45].

XGBoost is an optimized gradient-boosting decision tree method. The concept is to generate multiple decision tree models in a sequential manner, with each model generated to fit the residuals of the previous model and a regularization term used to control the complexity of each model, eventually combining all the decision trees generated to improve the accuracy of the prediction [46].

When constructing each ML model, the data were randomly divided into a training data set with 80% of the data and a test data set with 20% of the data. The training data set was used to perform hyperparameter tuning and validation of the model using a 10-fold cross-validation method. Then, the model with the best hyperparameter was selected as the final model, and information on the importance of the corresponding variable was obtained. Finally, the best model predictive performance of each ML method was evaluated with the test data set. To verify the accuracy of the models generated, the performance of each model was measured using four key evaluation metrics—symmetric mean absolute percentage error (SMAPE), relative absolute error (RAE), root relative squared error (RRSE), and root mean squared error (RMSE) (Table 2).

After constructing valid RF, SGB, Lasso, Ridge, and XGBoost predictive models, the fifth step involved obtaining the relative importance values generated by each method for each predictor variable/risk factor according to the converging ML model. The importance of the most and least important risk factors were 100 and 0, respectively.

In the sixth step, each ML method generated different importance values for each predictor variable since the different methods had individual characteristics. In order to integrate the advantages of these methods and obtain more stable results, the average importance value was used to integrate and compare the predictor variables that were more important overall in the set of importance rankings, thus, improving stability and completeness. In the seventh step, a final analysis was performed and the results discussed to obtain the final conclusion.

In order to construct accurate predictive semen count models, all predictive models were built with R version 3.6.2 and RStudio version 1.1.453 (http://www.R-project.org; accessed on 25 May 2022; https://www.rstudio.com/products/rstudio/; accessed on 25 May 2022). Each model was constructed using the associated software packages of R. RF, SGB, Lasso, Ridge, and XGBoost are available in the “ran-domForest” package version 4.7-1.1 [47], “gbm” package version 2.1.8 [48], “glmnet” package version 4.1-1 [49], and “XGBoost” package version 1.6.0.1 [50]. Finally, version 6.0-93 of the “caret” package was used to find the optimal hyperparameters for all models [51].

## 3. Results

We mainly targeted the younger health screening group for our study sample; therefore, the average age of the sample is relatively low (33.22 ± 4.36 years) and the descriptive statistics show that the study group consists of relatively young healthy and subhealth groups (Table 1). Although the study was a one-time semen analysis, through different ML algorithms, we were able to identify risk factors that may affect semen quality, which could contribute to the prevention of poor sperm quality in unmarried men. We used five ML techniques, RF, SGB, Lasso, Ridge, and XGBoost, to construct predictive models for sperm count. Each method was evaluated based on four performance indicators (SMAPE, RAE, RRSE, and RMSE); we found that the smaller the indicator, the better the predictive performance of the model. Table 3 provides the results of comparison of the predictive performance of the five models. Ridge shows the best performance for SMAPE (0.530) and RAE (0.964) and Lasso shows the best performance for RRSE (1.005) and RMSE (52.608).

Overall, although the predictive performance of the ML algorithms is slightly different, that of the five models is similar and excellent. The five ML methods use different concepts to obtain the variable importance of each risk factor. Therefore, we average the importance values generated by the five methods for the same risk factor and rank each risk factor in descending order of its average variable importance in order to integrate the variable importance information generated by the methods to obtain more robust results and to find the top 10 important risk factors for predicting sperm count.

**Figure 5:**
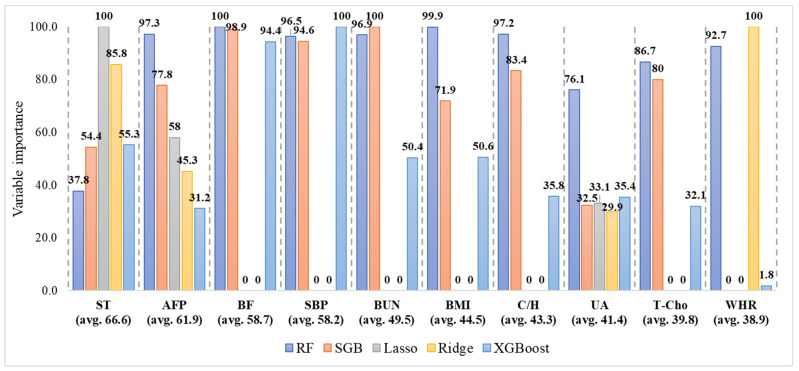
The variable importance to generated by the five algorithms for each risk factor. Note: ST: sleep time; AFP: alpha–fetoprotein; BF: body fat; SBP: systolic blood pressure; BUN: blood urea nitrogen; BMI: body mass index; C/H: T-Cho/HDL-C; UA: uric acid; T-Cho: total cholesterol; WHR: waist–hip ratio; RF: random forest; SGB: stochastic gradient boosting; Lasso: least absolute shrinkage and selection operator regression; Ridge: ridge regression; XGBoost: extreme gradient boosting.

Figure 5 illustrates the average top 10 risk factors in the five ML methods. The top ranked and most important risk factor is sleep time (ST) with an average importance of 66.6 (avg. 66.6). As mentioned before, the five ML methods generate different variable importance values for ST; RF generates a variable importance value of 37.8, SGB of 54.4, Lasso of 100, Ridge of 85.8, and XGBoost of 55.3. The average importance (the average of these five importance values) is 66.6. The second most important risk factor is AFP with an average importance of 61.9 (avg. 61.9). Similarly, the third to tenth important variables are BF, SBP, BUN, BMI, C/H, UA, T-Cho, and WHR, in that order.

To investigate the variables with greater clinical relevance, we focus on the top five important risk factors identified in this study, namely, ST, AFP, BF, SBP, and BUN.

## 4. Discussion

Both too-short and too-long sleep durations result in poor-quality semen [52]. Sleep disturbance is also associated with parameters indicating poor semen quality; men suffering from disturbed sleep show lower total sperm count, percentage of total and progressive motility, and percentage of morphologically normal spermatozoa compared to men enjoying high-quality sleep [53]. Sleep deprivation in rats increases stressful stimuli, which leads to the activation of the hypothalamus–pituitary–adrenal axis and causes elevated serum corticosteroid levels and decreased testosterone levels [54]. However, no difference in sperm count or sperm motility was found in this sleep-deprived animal model compared to the control groups. Therefore, whether sleep duration affects sperm quality through changing reproductive hormone levels or through different pathways affecting gene expression patterns related to spermatogenesis remains inconclusive.

Our study indicates that a shorter sleep duration has adverse effects on sperm count. It is possible that with a shorter sleep duration, reproductive hormone levels might be changed to a level that causes lower spermatogenesis. Further investigations into the link between sleep duration and sperm count are needed.

Alpha-fetoprotein is another risk factor identified by our established model. Few studies highlight this link between AFP and semen quality. In experiments with cryptorchid mice, AFP is specifically expressed in spermatocytes and secreted into the circulation [55]. Injection of AFP into the seminiferous tubules of normal mice could block spermiogenesis, the final step of spermatogenesis. A recent study found high serum AFP in male patients with aberrant sperm counts [56].

However, some of these studies were based on injecting AFP into the semen of animals, and the resulting concentration of AFP should be much higher than that found in healthy male patients. In our current study, we find a positive relationship between AFP and male sperm count. We suspect that there may be a U-shaped relationship between AFP and sperm count, meaning that both too low and too high levels have negative impacts on sperm count. However, it is still required for maintaining normal sperm count, and more studies are needed to illustrate its relationship with male sperm count.

BF, SBP, and other factors in our top 10 list of risk factors (BMI, C/H, T-Cho, and WHR) are related to metabolic syndrome, which has become a global epidemic. Metabolic syndrome has been linked to male infertility and poor semen quality [57], and many studies show that reproductive hormones are altered in males with the syndrome [58,59,60]. Our results support the view that more severe metabolic syndrome has an adverse effect on sperm count.

In the case of BUN, the fifth risk factor in our ranking, no investigations to date have been performed to find its direct link with male fertility or semen quality. However, chronic kidney disease (CKD) has been found to be associated with poor semen quality by affecting spermatogenesis and sperm motility [61]. The link between CKD and semen quality could be multifactorial. Most of these studies were based on the analysis of advanced CKD or patients under hemodialysis. However, in relatively healthy male patients, higher BUN levels seem to have a negative effect on sperm count. Therefore, the link between elevated BUN and sperm count in the healthy population or prior to the development of CKD requires further detailed study.

In summary, the established ML model successfully reproduces the findings of previous studies that sleep duration, BF, SBP, and BUN negatively affect sperm count. AFP is a lesser-known risk factor and more studies are needed to identify its relationship with male sperm count.

## 5. Limitations

This study was a cross-sectional study investigating the links between health examination data and sperm count of middle-aged males in Taiwan. The participants included 686 (49.89%) married and 619 (45.02%) unmarried males. Our study used five ML methods to analyze the risk factors affecting sperm count in healthy males. We listed these risk factors according to their importance in affecting sperm counts. Our study was based on a single analysis of semen; therefore, it does not truly reflect the participants’ fertility, which needs multiple analyses of semen at different time points. With enough participants, a cross-sectional study could more comprehensively identify risk factors linked to sperm count changes. In addition, ML enables the analysis of nonlinear relationships and complex interactions between multiple predictor variables in this study. However, the top five risk factors, except AFP, all have a negative impact on male sperm count. AFP shows a positive influence on male sperm count; however, there may be a U-shape relationship between AFP and sperm count. It is necessary for maintaining sperm count; however, both too much or too little can have adverse effects on sperm production. To support this hypothesis, more sophisticated algorithms are needed to identify these U-shaped relationships with sperm count.

## 6. Conclusions

From Taiwan’s health screening data of 1375 male patients, the established ML model predicts many risk factors affecting male semen qualities. Some of our predicted risk factors are consistent with previous results and thoroughly studied. Specially, ST is recognized in different algorithms and is the highest-ranking risk factor after sorting. After becoming a developed country, late marriage and low birth rate are important problems that need to be dealt with. Based on our studies and previous research, regular lifestyle and enough sleep duration are strongly suggested to improve semen quality and decrease the risk of male infertility indirectly.

The different algorithms in this study found sleep time to be the most important variable for predicting semen quality after joint ranking. Most residents of cities in developed countries, with a similar demographic and economic environment to that of Taiwan, tend to marry late and have fewer children. In view of the preliminary results of this study and its corroboration of findings of previous investigations, we suggest that the relevant government departments or health authorities in Taiwan should promote appropriate health information to the male population of reproductive age and advocate normal workloads and sufficient sleep and rest. This may help to avoid the risk of decreased sperm count or an indirect negative impact on male fertility.

## Figures and Tables

**Table 1:** The independent variables and the dependent variable analyzed in this study.

Independent Variable	N = 1375; n (%)	Independent Variable	N = 1375; n (%)
CS: Current smokers		ST: Sleep time (hours)	
(1) Never	911 (66.25%)	(1) <4	7 (0.51%)
(2) Passive smoking	56 (4.07%)	(2) 4–6	265 (19.27%)
(3) Quit	114 (8.29%)	(3) 6–7	811 (58.98%)
(4) Occasional	58 (4.22%)	(4) 7–8	248 (18.04%)
(5) Addicted	236 (17.16%)	(5) 8–9	44 (3.20%)
AD: Alcohol drinker		(6) >9	NA
(1) Never	1143 (83.13%)	MetS	
(2) Quit	17 (1.24%)	(1) No	1241 (90.25%)
(3) 1–2 times a week	169 (12.29%)	(2) Yes	134 (9.75%)
(4) 3–4 times a week	39 (2.84%)	**Independent Variable**	**Mean ± SD**
(5) 5–6 times a week	NA	Age	33.22 ± 4.36
(6) Addicted	7 (0.51%)	BMI (body mass index, kg/m^2^)	24.27 ± 3.37
Vitamin C supplementation		BF (body fat, %)	24.36 ± 5.57
(1) No	1156 (84.07%)	WC (waist circumference, cm)	82.26 ± 8.34
(2) Yes	219 (15.93%)	WHR (waist–hip ratio, %)	0.84 ± 0.05
Vitamin E supplementation		SBP (systolic blood pressure, mmHg)	118.22 ± 12.60
(1) No	1289 (93.75%)	DBP (diastolic blood pressure, mmHg)	72.99 ± 9.62
(2) Yes	86 (6.25%)	Hb (hemoglobin, g/dL)	15.22 ± 0.99
Consumption of Omega-3 rich food		FPG (fasting plasma glucose, mg/dL)	98.61 ± 10.60
(1) No	1283 (93.31%)	SGOT (serum glutamic oxaloacetic transaminase, U/L)	25.78 ± 20.02
(2) Yes	92 (6.69%)	SGPT (serum glutamic pyruvic transaminase, U/L)	36.97 ± 36.02
Consumption of sugar-containing beverages		BUN (blood urea nitrogen, mg/dL)	± 3.01
(1) No or less than 1 cup per week	356 (25.89%)	e-GFR (estimated glomerular filtration rate, ml/min/1.73m^2)^	± 11.23
(2) 1 to 3 cups per week	460 (33.45%)	UA (uric acid, mg/dL)	6.68 ± 1.27
(3) 4 to 6 cups per week	266 (19.35%)	TG (triglyceride, mg/dL)	118.3 ± 68.94
(4) 1 cup per day	198 (14.40%)	T-Cho (total cholesterol, mg/dl)	193.42 ± 32.54
(5) 2 or more than 2 cups per day	95 (6.91%)	HDL-C (high-density lipoprotein cholesterol, mg/dL)	52.36 ± 11.67
Daily physical activity		LDL-C (low-density lipoprotein cholesterol, mg/dL)	119.55 ± 30.63
(1) Sedentary most of the time	928 (67.49%)	C/H (T-Cho/HDL-C)	3.85 ± 0.96
(2) Frequent repeated sitting and ambulation	311 (22.62%)	AFP (alpha–fetoprotein, ng/mL)	2.74 ± 1.33
(3) Standing or ambulation most of the time	111 (8.07%)	**Dependent Variable**	**Mean ± SD**
(4) Requires whole body muscle usage most of the time	25 (1.82%)	S-C (sperm count)	53.3 ± 42.24

**Table 2:** Equations for calculating performance metrics.

Metric	Description	Calculation
SMAPE	Symmetric mean absolute percentage error	$\mathrm{SMAPE}=\frac{1}{n}\overset{n}{\underset{i=1}{\sum }}\frac{\left|y_{i}-{\hat{y}}_{i}\right|}{\left(\left|y_{i}\right|+\left|{\hat{y}}_{i}\right|\right)/2}\times 100$
RAE	Relative absolute error	$\mathrm{RAE}=\sqrt{\frac{\sum_{i=1}^{n}{\left(y_{i}-{\hat{y}}_{i}\right)}^{2}}{\sum_{i=1}^{n}{\left(y_{i}\right)}^{2}}}$
RRSE	Root relative squared error	$\mathrm{RRSE}=\sqrt{\frac{\sum_{i=1}^{n}{\left(y_{i}-{\hat{y}}_{i}\right)}^{2}}{\sum_{i=1}^{n}{\left(y_{i}-{\hat{y}}_{i}\right)}^{2}}}$
RMSE	Root mean squared error	$\mathrm{RMSE}=\sqrt{\frac{1}{n}\overset{n}{\underset{i=1}{\sum }}{\left(y_{i}-{\hat{y}}_{i}\right)}^{2}}$

${\hat{y}}_{i}$ and $y_{i}$ represent predicted and actual values, respectively; n stands for the number of instances.

**Table 3:** Model performance in predicting sperm count.

Methods	SMAPE	RAE	RRSE	RMSE
RF	0.537	0.984	1.014	53.060
SGB	0.536	0.977	1.017	53.218
Lasso	0.534	0.972	1.005	52.608
Ridge	0.530	0.964	1.006	52.674
XGBoost	0.532	0.968	1.011	52.913

Note: RF: random forest; SGB: stochastic gradient boosting; Lasso: least absolute shrinkage and selection operator regression; Ridge: ridge regression; XGBoost: extreme gradient boosting.

## Data Availability

All of the datasets collected from the MJ Health Research Foundation, the data need to apply and authorize the use, and the application procedures are accessed via this link. http://www.mjhrf.org/main/page/release1/en/#release01 (accessed on 1 October 2022).
